# Sugarcane Metabolome Compositional Stability in Pretreatment Processes for NMR Measurements

**DOI:** 10.3390/metabo12090862

**Published:** 2022-09-14

**Authors:** Yasuhiro Date, Chiaki Ishikawa, Makoto Umeda, Yusuke Tarumoto, Megumi Okubo, Yasuaki Tamura, Hiroshi Ono

**Affiliations:** 1 Research Center for Advanced Analysis, National Agriculture and Food Research Organization, Tsukuba 305-8642, Japan; 2 Institute of Food Research, National Agriculture and Food Research Organization, Tsukuba 305-8642, Japan; 3 Kyushu-Okinawa Agricultural Research Center, National Agriculture and Food Research Organization, Nishinoomote 891-3102, Japan

**Keywords:** metabolomics, nuclear magnetic resonance, sugarcane juice, metabolite composition, support vector machine, machine learning, harvest period

## Abstract

Sugarcane is essential for global sugar production and its compressed juice is a key raw material for industrial products. Sugarcane juice includes various metabolites with abundances and compositional balances influencing product qualities and functionalities. Therefore, understanding the characteristic features of the sugarcane metabolome is important. However, sugarcane compositional variability and stability, even in pretreatment processes for nuclear magnetic resonance (NMR)-based metabolomic studies, remains elusive. The objective of this study is to evaluate sugarcane juice metabolomic variability affected by centrifugation, filtration, and thermal pretreatments, as well as the time-course changes for determining optimal conditions for NMR-based metabolomic approach. The pretreatment processes left the metabolomic compositions unchanged, indicating that these pretreatments are compatible with one another and the studied metabolomes are comparable. The thermal processing provided stability to the metabolome for more than 32 h at room temperature. Based on the determined analytical conditions, we conducted an NMR-based metabolomic study to discriminate the differences in the harvest period and allowed for successfully identifying the characteristic metabolome. Our findings denote that NMR-based sugarcane metabolomics enable us to provide an opportunity to collect a massive amount of data upon collaboration between multiple researchers, resulting in the rapid construction of useful databases for both research purposes and industrial use.

## 1. Introduction

Sugarcane is an indispensable crop for global sugar production and is used as feedstock for sugar, bioethanol, and other industrial products. Sugarcane is typically cultivated in tropical and subtropical regions in countries such as Brazil, India, and China. In Japan, sugarcane is commercially produced on the Nansei islands located in Okinawa and Kagoshima prefectures where sugarcane cannot be replaced with other crops [1]. Therefore, sugarcane plays a pivotal role in the economy, as well as the social and sustainable development in the Nansei islands [2].

Although sugarcane improvement techniques are important for an efficient crop, feedstock, and industrial product production and quality improvements, it is hampered by several factors such as higher polyploidy and a longer production cycle [3]. To tackle this obstacle, omics approaches, including metabolomics, are potential biotechnological interventions to improve sugarcane quality and productivity [4]. Metabolomics is an approach for the comprehensive detection and analysis of various metabolites produced by the complex metabolic reactions in biological systems. Nuclear magnetic resonance (NMR), a key technique in the metabolomics, exhibits several advantages such as high reproducibility, noninvasive measurement approach, and interinstitutional compatibility [5]. NMR-based metabolomics is applied in various research fields including food science and nutrition research [6,7].

In sugarcane research, the NMR-based metabolomic approach was successfully applied for metabolite composition profiling, genotype discrimination, and metabolic biomarker discovery. For example, Mahmud et al. reported distinct metabolomic profiles between embryogenic and nonembryogenic callus tissues [8] and revealed the biochemical relationship between callus tissues and their culture media [9]. Sabino et al. demonstrated higher chlorogenic acid accumulation in sugarcane leaves and its function as a natural biopesticide in response to *Diatraea saccharalis* [10]. Using a combined approach of NMR and mass spectrometry, Coutinho et al. explored metabolite biomarkers for a breeding program of sugarcane cultivars in Brazil [11]. Ali et al. reported the metabolomic profiles and antioxidant activity of sugarcane juice and its byproduct molasses [12]. In this sense, NMR-based metabolomics is a powerful tool to analyze the sugarcane metabolome and is a potential assistance tool for sugarcane quality and productivity improvement.

Sugarcane juice is a key raw material for industrial products such as refined sugar, brown sugar, or molasses, and it is also consumed as a beverage. Sugarcane juice is also used in the treatment of urinary diseases [13]. Sugarcane juice includes not only sucrose but also various metabolites such as amino and organic acids, and their abundances and compositional balances involve the product qualities and functionalities. Therefore, understanding characteristic metabolomic features and compositional variabilities in sugarcane juice is important for quality control and consistent production in the manufacturing processes. Moreover, sugarcane juice is a perishable product. However, little is known about the compositional variability and stability of not a specific sugarcane metabolite, such as sucrose, but the entire metabolome.

This study focused on the evaluation of metabolomic profiles and compositional variabilities in sugarcane juice using an NMR-based metabolomic approach. Since little information was available on how the NMR measurement pretreatment processes could impact the metabolomic profiles, we evaluated the compositional variabilities during the centrifugation, filtration, and thermal treatment processes for determining optimal conditions to perform NMR-based metabolomic approach targeted for sugarcane juice samples. Time-course variations at room temperature were also evaluated to determine how long the metabolome would remain stable and unchanged. Based on the obtained analytical chemical information, an NMR-based metabolomic approach was used to discriminate the metabolomic differences during the harvest period to capture the trend and characteristics in sugarcane juice compositional variabilities.

## 2. Materials and Methods

### 2.1. Plant Material

Sugarcane samples were cultivated at an agricultural field in the Tanegashima Research Station, Kyushu-Okinawa Agricultural Research Center, NARO, Japan (30°43′ N, 131°04′ E). The soil type in the field was Silandic Andosols. The agricultural filed has gentle slope on east side with an altitude of 45 m. Average annual temperature and annual amount of precipitation in this area is 19.6 °C and 2322 mm, respectively. The sugarcanes used in this study included a total of 562 samples of 6 varieties (Harunoogi [14] (see also the related website, https://www.jircas.go.jp/sites/default/files/seika/2019/2019_B06_A4_en.pdf) (accessed on 1 August 2022), KTn03-54 [15], NCo310, Ni22, NiF8, and NiTn18) and dozens of selected clones collected from the 4th selection, regional adaptability test, and local yield evaluation in the sugarcane breeding program in Japan [1]. The sugarcane sets were planted and the grown stalks were harvested in April and upcoming January, respectively. First ratoon canes sprouted from the plant canes were harvested mainly in November (several samples were harvested in early December). Second ratoon canes sprouted from the first ratoon canes were harvested in November of the following year. These cultivations were repeated for three years between 2018 and 2020. Chemical fertilizer was applied three times a year as one basal (7.2 g N m^−^^2^, 12.0 g P_2_O_5_ m^−2^, 6.0 g K_2_O m^−2^) and two top (4.5 g N m^−2^, 4.5 g K_2_O m^−2^) dressings. Irrigation was not applied for the sugarcane cultivation.

### 2.2. Sample Preparation

The head part of sugarcanes was removed from the stalk part. We then finely crushed 10 whole stalk parts using a shredder (KS-MS, Matsuo, Kagoshima, Japan) and subsequently pressed 500 g of the finely crushed stalk using a Hotpress machine (CSS-NP-02H, Nittoku, Chiba, Japan). The obtained sugarcane juice was located on ice within 30 min and stored at −20 °C within several hours until further analysis.

### 2.3. Preparation for NMR Measurements

The sugarcane juice samples were immediately pretreated after thawing with shaking at 1400 rpm for 10 min using a ThermoMixer comfort (Eppendorf GmbH, Hamburg, Germany) at constant temperatures (55 °C, 65 °C, 75 °C, 85 °C, or 95 °C) to evaluate how thermal treatment affects metabolite compositions. The samples were then centrifuged at 4 °C and 15,000 rpm (20,630× *g*) for 10 min. To evaluate the filtration effects and metabolomic characterization for the sugarcane harvest period, the sugarcane juice samples were filtrated with a filter aid (super light 4–7 µm, Tokyokonno Co. Ltd., Tokyo, Japan). All samples were mixed with a phosphate-buffered solution (100 mM K_2_HPO_4_/KH_2_PO_4_ and 10% deuterium oxide, pH 7.4) including an internal standard for NMR spectroscopy (1 mM sodium trimethylsilylpropanesulfonate (DSS)-*d*_6_).

### 2.4. NMR Measurements

All NMR spectra obtained in this study were recorded on a Bruker Avance III HD 700 NMR spectrometer (Bruker BioSpin GmbH, Rheinstetten, Germany) equipped with a 5 mm Cryo TCI probe and an autosampler (SampleJet, Bruker BioSpin GmbH, Rheinstetten, Germany). For the metabolomic analysis, two-dimensional *J*-resolved NMR measurements were performed at 298 K using a slightly modified version of the Bruker standard pulse program “jresgpprqf” with the following acquisition parameters; data points, 32 for F1 and 16 K for F2; number of scans, 8; number of dummy scans, 16; spectral widths, 43.8 Hz for F1 and 11 ppm for F2. The obtained spectra were preprocessed using the TopSpin software version 4.1.1 (Bruker BioSpin GmbH, Rheinstetten, Germany) and converted into pseudo-one-dimensional ^1^H NMR spectra by skyline projection. The spectra were subsequently processed by rNMR [16] running on the R software version 3.6.1 (R core team, Vienna, Austria) [17] in order to convert the NMR signals into numerical values based on the regions of interest (ROIs).

### 2.5. Data Analysis

The ROIs derived from and largely affected by sucrose, DSS, and the solvent were excluded from further analyses. The dataset was normalized based on the signal intensity of the DSS internal standard. The support vector machine (SVM) was performed using an e1071 package coupled with a mean decrease accuracy method reported in a previous study [18] running on RStudio desktop open source edition version 1.4.1717 (R version 4.0.2 (R core team, Vienna, Austria)) [19]. The model construction, hyperparameter optimization, and validation were performed by a repeated (n = 10) 3- and 5-fold double (nested) cross-validation. The receiver operating characteristic (ROC) curve and the area under the curve (AUC) were analyzed using an ROCR package. The metabolite annotations were performed using the SpinCouple program [20] combined with validation by reference to the Human Metabolome Database version 5.0 (https://hmdb.ca/) (accessed on 5 July 2022) [21]. Significant differences were calculated using Welch’s *t* test.

## 3. Results

### 3.1. Metabolomic Evaluation of Compositional Variability in Sugarcane Juice

Since sugarcane juice is well-known to be perishable, we assumed that its chemical composition might be changeable and unstable. Therefore, we evaluated if the NMR measurement pretreatment could induce variability and stability in the sugarcane juice chemical composition before evaluating the sugarcane harvest period-related metabolomic profiles using our NMR-based metabolomic approach. For this analysis, we used the juice of the “NiF8” sugarcane variety collected from an agricultural field in the Tanegashima Research Station.

#### 3.1.1. Filtration and Centrifugation Process-Related Effect

**Figure 1:**
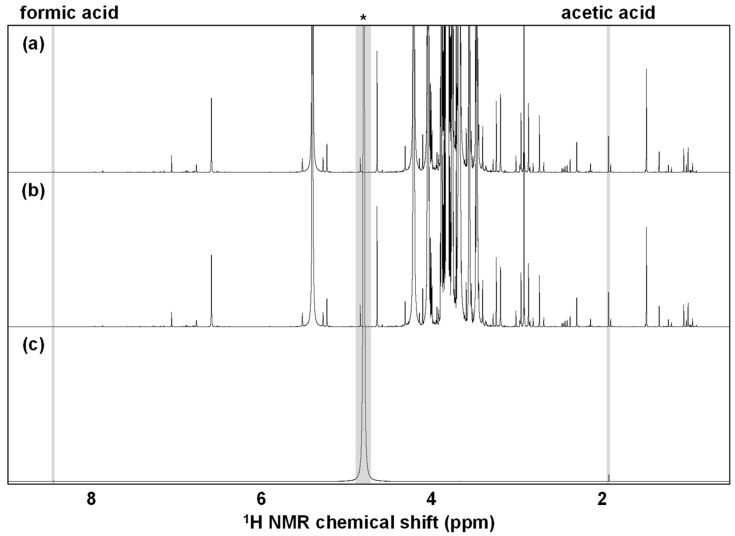
Representative ^1^H NMR spectra of sugarcane juice samples pretreated by filtration with filter aid (**a**) or centrifugation (**b**). Contaminants from the filter or filter aid in the filtration process could also be observed on the NMR spectrum with no sugarcane juice sample (**c**). *, solvent: water.

In general, the elimination of suspended solids in a sample solution is preferred for solution-state NMR measurements. To eliminate any turbid materials in the sugarcane juice samples, we performed filtration using a filter aid or centrifugation at 15,000 rpm for 10 min. The comparison of the filtration and the centrifugation processes indicated that the filtered sample solution treated was clearer than the centrifuged one, while little difference could be observed between them in terms of signal pattern and intensities in the NMR spectra (Figure 1). The NMR spectrum of the filtrated sample with no sugarcane juice exhibited slight contamination, a small peak derived from acetic acid, and a very small peak derived from formic acid using the filter or filter aid in the filtration process. This contamination barely affected the NMR spectra due to the very small intensities of the contaminants compared to the sugarcane juice components.

#### 3.1.2. Thermal Treatment-Related Effect

**Figure 2:**
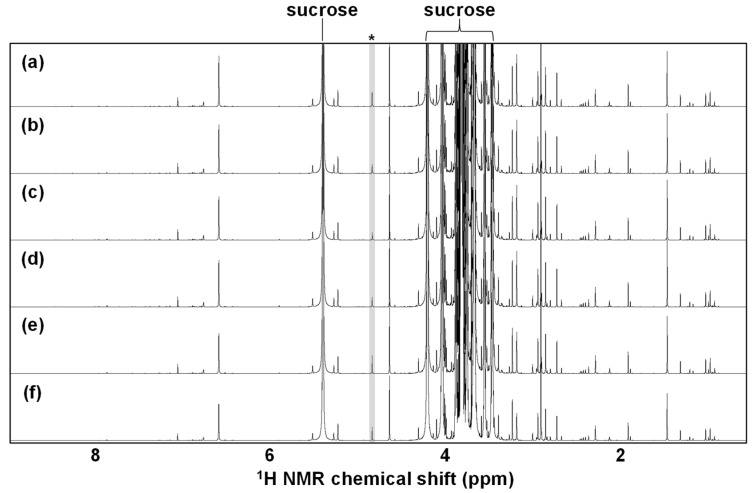
^1^H NMR spectra of sugarcane juice samples heated at constant temperatures set to 95 °C (**a**), 85 °C (**b**), 75 °C (**c**), 65 °C (**d**), or 55 °C (**e**). An NMR spectrum with no thermal treatment is also presented as a control (**f**). *, solvent: water.

Thermal treatment is often applied as a pretreatment step for NMR measurements in metabolomic studies. The thermal treatment conditions are different with respect to each previous study (e.g., the temperatures set to 55 °C [22] or 90 °C [23]). To evaluate how thermal treatment affects the NMR spectra, the sugarcane juice samples were heated for 10 min at constant temperatures set to 55 °C, 65 °C, 75 °C, 85 °C, or 95 °C. The signal pattern and intensities in the NMR spectra were almost identical with or without a series of thermal treatments (Figure 2). This result indicated that the thermal treatments affected hardly the alteration of the metabolite compositions in the sugarcane juice samples, i.e., the metabolome was thermally stable.

#### 3.1.3. Metabolite Alterations during the NMR Measurement

**Figure 3:**
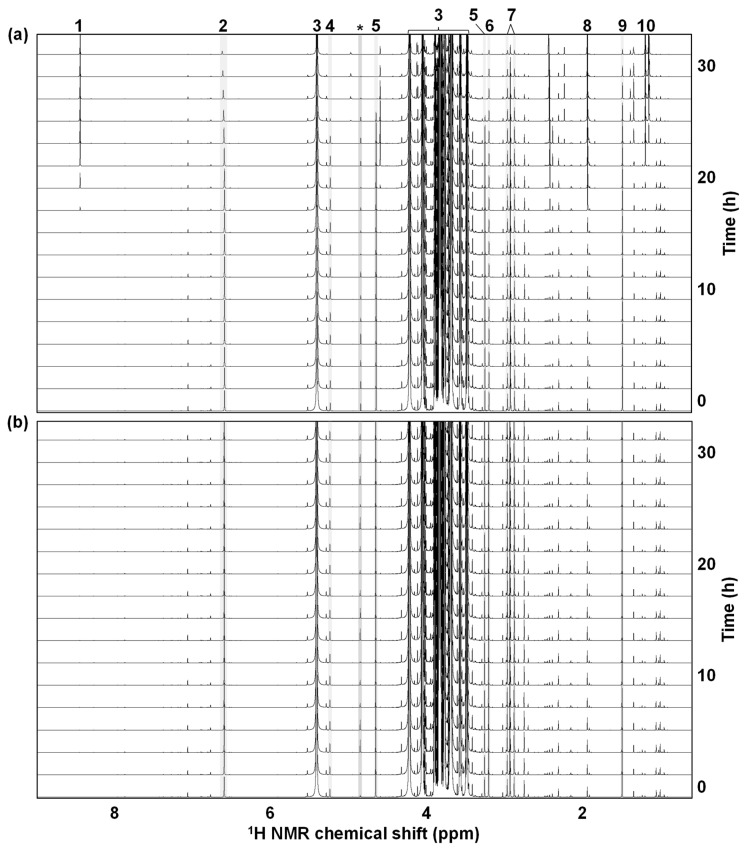
Time-course changes of metabolite compositions in sugarcane juice during the NMR measurements. The samples were processed with no thermal treatment (**a**) or thermal treatment at 95 °C for 10 min (**b**). 1, formic acid; 2, *trans*-aconitic acid; 3, sucrose; 4, α-glucose; 5, β-glucose; 6, choline; 7, asparagine; 8, acetic acid; 9, alanine; 10, ethanol; *, solvent: water.

Although the perishable nature of sugarcane juice leads to time-course-related changes in its metabolite composition, the timing and impact of metabolite degradation on the NMR spectrum remain unclear. We thus evaluated the metabolite composition stability and deterioration at room temperature by continuous measurements of the sugarcane juice samples in an NMR machine. The NMR spectra unraveled that the compositional alterations of the metabolites could be observed approximately 15 h after the start of the experiment, accompanied by the degradation of sugar and amino acid metabolites such as glucose, alanine, and asparagine, and the production of organic acid and alcohol metabolites such as acetic acid, formic acid, and ethanol (Figure 3a). Interestingly, a metabolomic profile remained unchanged for the experimental period (approximately 32 h) in the case of the sample processed at the thermal treatment of 95 °C for 10 min (Figure 3b).

Taken together, the signal pattern and intensities in the NMR spectra were rarely affected by the pretreatment processes such as filtration, centrifugation, and thermal treatment. Therefore, due to its relatively easy handling, we adopted filtration with filter aid as an NMR measurement pretreatment step for the subsequent metabolomic analysis.

### 3.2. Metabolomic Characterization Based on the Harvest Period-Related Differences

In Tanegashima Island, plant and ratoon canes are harvested in January and November, respectively. Since sugarcane juice metabolite compositions influence qualities of their industrial products, understanding the harvest period-related differences, trends, and characteristics of the sugarcane metabolome is important for feedstock quality control. Therefore, we performed the metabolomic characterization of sugarcane juice based on differences of harvest period using an NMR-based metabolomic approach.

#### 3.2.1. Sugarcane Harvest Period Classification Model

**Figure 4:**
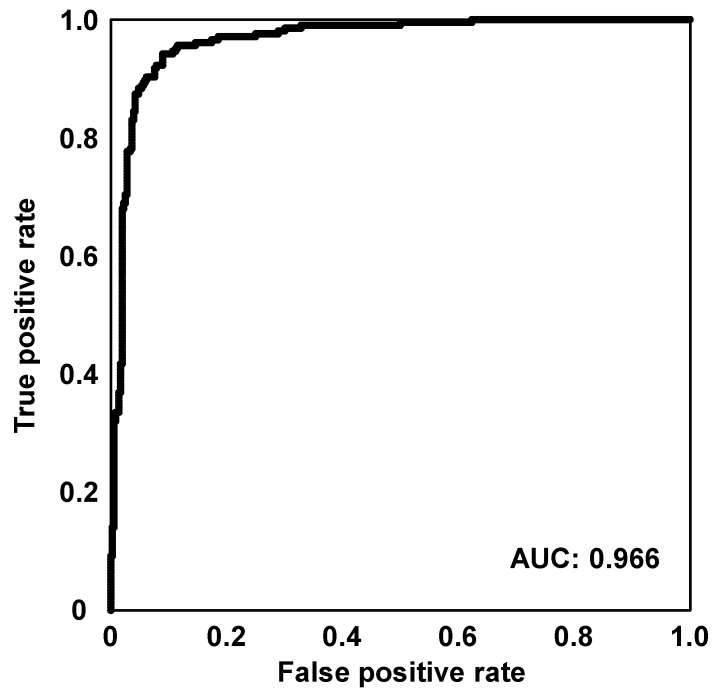
ROC curve of the constructed SVM model for harvest period classification.

The metabolomic characterization was conducted using 562 sugarcane samples including 6 varieties [1] harvested in November (first and second ratoon canes) and January (plant canes) collected from an agricultural field in the Tanegashima Research Station (Table S1). To maximize the metabolomic differences between the harvest periods, we adopted a SVM for classification model construction as the SVM exhibits relatively good performance in the case of small sample size [18]. The SVM model was constructed using 3-fold cross-validation for hyperparameter optimization nested in repeated (n = 10) 5-fold cross-validation for test data evaluation. The constructed SVM model displayed a good classification performance (Table 1) with an accuracy of 95.3%. To verify the constructed SVM model for harvest period classification, we calculated the ROC curve and the AUC (Figure 4). The AUC value was 0.966, indicating that the constructed SVM model was reliable.

#### 3.2.2. Important Metabolites Contributed to the Harvest Period Discrimination

**Figure 5:**
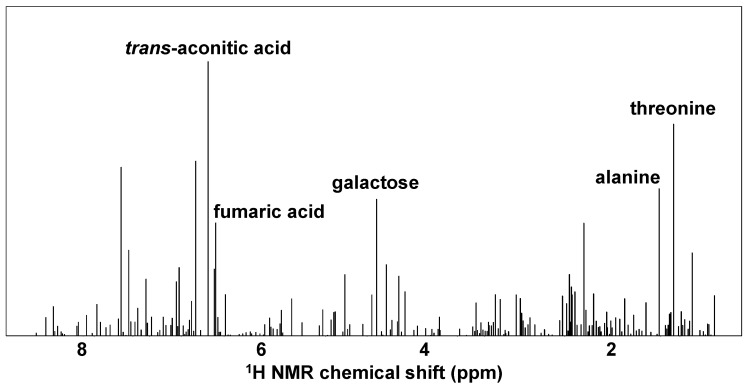
Important metabolites contributing to the harvest period-related SVM classification model.

**Figure 6:**
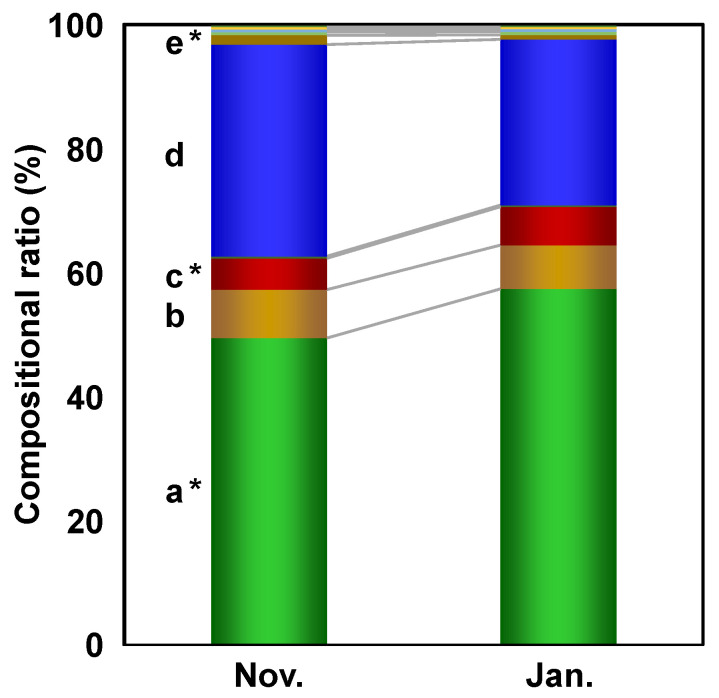
Compositional ratios of top 10 metabolites identified by the analysis of importance. a, *trans*-aconitic acid; b, threonine; c, unannotated metabolite; d, alanine; e, galactose; *, *p* < 0.01.

The SVM classification model construction was accompanied by the calculation of important variables contributing to the discrimination of the harvest periods. The calculation was performed by the mean decrease accuracy method described in a previous study [18]. The calculation results identified several compounds, such as *trans*-aconitic acid, threonine, alanine, and galactose, as characteristic metabolites in discriminating the differences between the harvest periods (Figure 5). The compositional balances of the top 10 metabolites identified by the analysis of importance were different between the sugarcane juice samples harvested in November and January (Figure 6). In particular, the compositional ratio of *trans*-aconitic acid was likely to be relatively high in January compared to that in November, whereas the same ratio of alanine was likely to be relatively low.

## 4. Discussion

This study focused on the evaluation of compositional variabilities upon sugarcane juice sample pretreatment processes for NMR measurements and the characterization of metabolomic profiles based on the harvest periods. The metabolite compositions remained unchanged upon pretreatment processes with or without centrifugation, filtration, and thermal treatments conducted in this study. This result denotes that these pretreatment processes are compatible with one another, and the metabolomic profiles are comparable in NMR-based metabolomic studies. Considering the NMR-based metabolomic data comparability among different laboratories and NMR machines [5], the stability and robustness of the metabolomic profiling allow for the analysis of the own data of the researchers combined with data obtained by a third party. In addition, the high-throughput NMR-based metabolomic approach performance [24] should promote a massive amount of data collection in sugarcane metabolomics by the collaboration of multiple researchers, resulting in the rapid construction of useful databases for both research purposes and industrial use. Therefore, NMR-based sugarcane metabolomics should be a helpful approach adapted to the era of big data.

In this study, only a specific filter was used for evaluation of filtration process-related effect. Therefore, several effects such as contamination and adsorption might be caused when the other filter types and materials were used for filtration. Moreover, although our results indicated comparability in NMR-based metabolomic data affected by pretreatment processes conducted in this study, it is still controversial whether the same conclusion is obtained in terms of not only sugarcane juice but also sugarcane-derived materials and products such as leaves and molasses.

The time-course incubation at room temperature demonstrated hardly altered metabolite compositions for approximately 15 h despite the perishable nature of sugarcane juice, indicating that sugarcane juice remained relatively more stable at room temperature than expected regarding the detectable metabolites and their composition using the NMR-based metabolomic approach. The metabolite alterations after 15 h demonstrated amino acid and sugar consumption with organic acid and ethanol production, suggesting that the metabolite components were degraded by the microbial community of the sugarcane juice samples. In addition, the thermal processing of the sugarcane samples resulted in more stable metabolite compositions or unchanged metabolomic profiles for more than 32 h. Therefore, we recommend thermally pretreating sugarcane samples for NMR measurements when metabolically stable samples are required.

The NMR-based metabolomic approach allowed for capturing the compositional differences between the harvest periods in November (ratoon canes) and January (plant canes). Abundances and compositional balances of the metabolome, such as in the case of *trans*-aconitic acid, threonine, and alanine, were the major characteristic differences between the harvest periods (and cultivation types) although further investigation was required to elucidate the significance of the metabolome in crop productions and the effects on product qualities and functionalities. This study is a first step in establishing a database of the sugarcane metabolome related to not only harvest periods and cultivation types but also various characteristic phenotypes such as varieties, growth stages, and geographical origins using an NMR-based metabolomic approach. The establishment of the sugarcane metabolome coupled with the phenotypic information in the database should involve various challenges such as further acceleration of research and development, efficient improvement of sugarcane crop production and quality, quality control optimization, and consistent production during the manufacturing processes by the effective use of metabolomic big data. More specifically, for example, the metabolomic approach provides a possibility for discovering metabolite biomarkers from specific characteristic phenotype-based metabolite differentiation, e.g., high disease resistance and tolerance to low temperatures. At the moment, the discovered biomarkers should have significant potential as indicators for improving sugarcane qualities and optimizing sugarcane cultivation adapted to low temperatures in a given region. Therefore, NMR-based metabolomic approaches combined with big data should provide accelerative and efficient crop improvement and production with high added value, leading to the sustainable development of the local economy and society in the future.

## 5. Conclusions

In this study, we evaluated sugarcane juice metabolomic variability affected by centrifugation, filtration, and thermal pretreatments for determining optimal conditions to perform NMR-based metabolomic approach. The results indicated that the pretreatment processes conducted in this study left the metabolomic compositions unchanged, indicating that these pretreatments are compatible with one another and the studied metabolomes are comparable in NMR-based metabolomic studies. The thermal processing of the sugarcane samples resulted in more stable metabolite compositions for more than 32 h at room temperature. The NMR-based metabolomic approach allowed for capturing the compositional differences between the harvest periods in November and January and successfully identifying the characteristic metabolome such as *trans*-aconitic acid, threonine, and alanine. This study is a first step in establishing a database of the sugarcane metabolome related to various characteristic phenotypes such as harvest periods, cultivation types, varieties, growth stages, and geographical origins using an NMR-based metabolomic approach. Our findings denote that NMR-based sugarcane metabolomics enable us to provide an opportunity to collect a massive amount of data upon collaboration between multiple researchers, resulting in the rapid construction of useful databases for both research purposes and industrial use.

## Figures and Tables

**Table 1:** Confusion matrix of the constructed SVM model for harvest period classification.

Predict\Correct	November	January
**November**	334.3	21.7
**January**	21.7	184.3

The displayed values represent the average (n = 10).

## Data Availability

The data presented in this study are available in Supplementary Material.

## Supplementary Materials

The following supporting information can be downloaded at: https://www.mdpi.com/article/10.3390/metabo12090862/s1, Table S1: The dataset used in this study.

## References

[1] Terajima Y., Hattori T., Shimatani M., Sato M., Takaragawa H., Sakaigaichi T., Umeda M., Naito T., Irei S. (2022). Sugarcane breeding and supporting genetics research in Japan. Sugar Tech..

[2] Matsuoka M. (2006). Sugarcane cultivation and sugar industry in Japan. Sugar Tech..

[3] Mustafa G., Joyia F.A., Anwar S., Parvaiz A., Khan M.S. (2018). Biotechnological interventions for the improvement of sugarcane crop and sugar production. Sugarcane—Technology and Research.

[4] Ali A., Khan M., Sharif R., Mujtaba M., Gao S.J. (2019). Sugarcane Omics: An Update on the Current Status of Research and Crop Improvement. Plants.

[5] Ward J.L., Baker J.M., Miller S.J., Deborde C., Maucourt M., Biais B., Rolin D., Moing A., Moco S., Vervoort J. (2010). An inter-laboratory comparison demonstrates that [H]-NMR metabolite fingerprinting is a robust technique for collaborative plant metabolomic data collection. Metabolomics.

[6] Consonni R., Cagliani L.R. (2019). The potentiality of NMR-based metabolomics in food science and food authentication assessment. Magn. Reson. Chem..

[7] Brennan L. (2014). NMR-based metabolomics: From sample preparation to applications in nutrition research. Prog. Nucl. Magn. Reson. Spectrosc..

[8] Mahmud I., Shrestha B., Boroujerdi A., Chowdhury K. (2015). NMR-based metabolomics profile comparisons to distinguish between embryogenic and non-embryogenic callus tissue of sugarcane at the biochemical level. Vitr. Cell. Dev. Biol. Plant.

[9] Mahmud I., Thapaliya M., Boroujerdi A., Chowdhury K. (2014). NMR-based metabolomics study of the biochemical relationship between sugarcane callus tissues and their respective nutrient culture media. Anal. Bioanal. Chem..

[10] Sabino A.R., Tavares S.S., Riffel A., Li J.V., Oliveira D.J., Feres C.I., Henrique L., Oliveira J.S., Correia G.D., Sabino A.R. (2019). 1H NMR metabolomic approach reveals chlorogenic acid as a response of sugarcane induced by exposure to Diatraea saccharalis. Ind. Crops Prod..

[11] Coutinho I.D., Baker J.M., Ward J.L., Beale M.H., Creste S., Cavalheiro A.J. (2016). Metabolite Profiling of Sugarcane Genotypes and Identification of Flavonoid Glycosides and Phenolic Acids. J. Agric. Food Chem..

[12] Ali S.E., El Gedaily R.A., Mocan A., Farag M.A., El-Seedi H.R. (2019). Profiling Metabolites and Biological Activities of Sugarcane (Saccharum officinarum Linn.) Juice and its Product Molasses via a Multiplex Metabolomics Approach. Molecules.

[13] Singh A., Lal U.R., Mukhtar H.M., Singh P.S., Shah G., Dhawan R.K. (2015). Phytochemical profile of sugarcane and its potential health aspects. Pharmacogn. Rev..

[14] Hattori T., Terajima Y., Sakaigaichi T., Terauchi T., Tarumoto Y., Adachi K., Hayano M., Tanaka M., Ishikawa S., Umeda M. (2019). High Ratoon Yield Sugarcane Cultivar “Harunoogi” Developed for Kumage Region by Using an Interspecific Hybrid between a Commercial Cultivar and Saccharum spontaneum L.. J. NARO Res. Dev..

[15] Sakaigaichi T., Terauchi T., Matsuoka M., Terajima Y., Hattori T., Irei S., Ujihara K., Sugimoto A., Ishikawa S., Tanaka M. (2017). Stalk Weight Type Sugarcane Variety “KTn03-54” with Early Maturing in the Kumage Region of Kagoshima Prefecture. Bull. NARO Agric. Res. Kyushu Okinawa Reg..

[16] Lewis I.A., Schommer S.C., Markley J.L. (2009). rNMR: Open source software for identifying and quantifying metabolites in NMR spectra. Magn. Reson. Chem..

[17] R Core Team (2019). R: A Language and Environment for Statistical Computing.

[18] Date Y., Kikuchi J. (2018). Application of a Deep Neural Network to Metabolomics Studies and Its Performance in Determining Important Variables. Anal. Chem..

[19] R Studio Team (2021). R Studio: Integrated Development Environment for R.

[20] Kikuchi J., Tsuboi Y., Komatsu K., Gomi M., Chikayama E., Date Y. (2016). SpinCouple: Development of a Web Tool for Analyzing Metabolite Mixtures via Two-Dimensional J-Resolved NMR Database. Anal. Chem..

[21] Wishart D.S., Guo A., Oler E., Wang F., Anjum A., Peters H., Dizon R., Sayeeda Z., Tian S., Lee B.L. (2022). HMDB 5.0: The Human Metabolome Database for 2022. Nucleic. Acids. Res..

[22] Ogura T., Date Y., Masukujane M., Coetzee T., Akashi K., Kikuchi J. (2016). Improvement of physical, chemical, and biological properties of aridisol from Botswana by the incorporation of torrefied biomass. Sci. Rep..

[23] Sekiyama Y., Okazaki K., Kikuchi J., Ikeda S. (2017). NMR-Based Metabolic Profiling of Field-Grown Leaves from Sugar Beet Plants Harbouring Different Levels of Resistance to Cercospora Leaf Spot Disease. Metabolites.

[24] Vignoli A., Ghini V., Meoni G., Licari C., Takis P.G., Tenori L., Turano P., Luchinat C. (2019). High-Throughput Metabolomics by 1D NMR. Angew. Chem. Int. Ed. Engl..

